# Coherent Precipitation and Strengthening in Compositionally Complex Alloys: A Review

**DOI:** 10.3390/e20110878

**Published:** 2018-11-15

**Authors:** Qing Wang, Zhen Li, Shujie Pang, Xiaona Li, Chuang Dong, Peter K. Liaw

**Affiliations:** 1 Key Laboratory of Materials Modification by Laser, Ion and Electron Beams (Ministry of Education), School of Materials Science and Engineering, Dalian University of Technology, Dalian 116024, China; 2 School of Mechanical Engineering, Dalian University of Technology, Dalian 116024, China; 3 Key Laboratory of Aerospace Materials and Performance (Ministry of Education), School of Materials Science and Engineering, Beihang University, Beijing 100191, China; 4 Department of Materials Science and Engineering, The University of Tennessee, Knoxville, TN 37996, USA

**Keywords:** precipitation, strengthening, coherent microstructure, conventional alloys, high entropy alloys

## Abstract

High-performance conventional engineering materials (including Al alloys, Mg alloys, Cu alloys, stainless steels, Ni superalloys, etc.) and newly-developed high entropy alloys are all compositionally-complex alloys (CCAs). In these CCA systems, the second-phase particles are generally precipitated in their solid-solution matrix, in which the precipitates are diverse and can result in different strengthening effects. The present work aims at generalizing the precipitation behavior and precipitation strengthening in CCAs comprehensively. First of all, the morphology evolution of second-phase particles and precipitation strengthening mechanisms are introduced. Then, the precipitation behaviors in diverse CCA systems are illustrated, especially the coherent precipitation. The relationship between the particle morphology and strengthening effectiveness is discussed. It is addressed that the challenge in the future is to design the stable coherent microstructure in different solid-solution matrices, which will be the most effective approach for the enhancement of alloy strength.

## 1. Introduction

Precipitation strengthening with intermetallic compounds is the most effective approach for the enhancement of alloy strength in engineering structural materials, compared with solid-solution strengthening, grain-boundary strengthening, and work hardening [1,2,3]. Especially at high temperatures (HTs), the precipitation strengthening is indispensable due to the prominent long-time microstructural stabilities caused by second-phase precipitates in the solid-solution matrix [4,5,6,7,8]. Among them, the coherent ordered phases, such as L1_2_-Ni_3_Al (*cP*4-Cu_3_Au) of the face-centered-cubic (FCC) solid solution [4,5], and B2-NiAl (*cP*2-ClCs) of the body-centered-cubic (BCC) solid solution [9,10,11,12,13], are crucial for the HT creep-resistant properties of alloys due to the perfect coherency between the ordered phase and the solid-solution matrix. It should be pointed out that the precipitation strengthening is related not only to the macroscopic properties of precipitates, but also to their microstructural morphologies. For instance, the prominent creep-resistant property of Ni-based superalloys at up to 85% of the insipient melting temperature (as high as 1100 °C) is primarily attributed to the special microstructure of spherical or cuboidal L1_2_-Ni_3_Al nanoprecipitates coherently-precipitated into the FCC matrix [5]. 

In order to meet the service-performance requirements, including mechanical strength, corrosion- and oxidation-resistant properties, etc., several solute elements are generally added to alloy or minor-alloy the solvent matrix constituted of one or two primary elements in conventional engineering structural materials [14]. From the viewpoint of element species, most of high-performance metallic materials, including Al alloys, Mg alloys, Cu alloys, stainless steels, and Ni superalloys, are all compositionally-complex alloys (CCAs), resulting in a uniform microstructure of diverse second-phase particles distributed in their solid-solution matrix. Recently, another kind of newly-developed CCAs are not based on one or two solvent elements, but based on the equimolar or near-equimolar mixing of multi-principal elements, which are also named high-entropy alloys (HEAs) [15,16,17,18,19,20]. HEAs have attracted more attention due to their unique properties resulted from simple crystalline structures, such as FCC, BCC, close-packed hexagonal (HCP), and their derivatives (L1_2_, B2, etc.) [19,20,21,22]. Thus, they can also be regarded as a special kind of solid-solution alloys, similar to conventional engineering alloys.

Therefore, the present work will comprehensively generalize the precipitation behavior and precipitation strengthening in CCAs, including conventional engineering alloys and high-entropy alloys, where the coherent precipitation will be specially emphasized. The morphology evolution of second-phase particles and precipitation strengthening mechanisms will be illustrated firstly. Then, the relationship between the particle morphology (shape and particle size) and strengthening effectiveness in diverse CCAs will be discussed, respectively. Finally, several thoughts on the coherent precipitation to design and develop high-performance CCAs in the future will be suggested.

## 2. Equilibrium Morphology of a Misfitting Particle

In the absence of elastic stress, the equilibrium morphology of a second-phase particle embedded into a matrix is established solely by the particle-matrix interfacial energy and its dependence on crystallographic orientation [23]. However, experimentally, the particle morphology, which arises during a diffusional phase transition in many alloys, was often not the shape that minimizes the total interfacial energy. In most cases, the presence of the lattice misfit between the second phase and the matrix can induce an elastic stress field, which in turn, affects the particle morphology [24]. For instance, in Ni-based superalloys with a microstructure of ordered L1_2_-Ni_3_Al particles coherently-embedded into the FCC solid-solution matrix, the particles were observed to undergo changes in shape from spheres to cuboids, and then to plates with increasing particle size [25,26], or even fission into smaller particles once they reach a critical size [27,28,29]. Actually, the particle equilibrium shape is determined by minimizing the total energy *E_t_*, the sum of interfacial energy, *E_i_*, and elastic energy, *E_e_*, at a constant particle volume, in which the *E_i_* and *E_e_* scale with the surface area and the volume of the particle, respectively [30,31]. So, the equilibrium shape of a misfitting particle is dependent on the particle size. That is to say, the particle shape should tend towards the shape that minimizes the *E_i_* at a smaller particle size, and towards the shape that minimizes the *E_e_* at a larger size. Furthermore, with increasing particle size, the elastic energy plays an increasingly important role in setting the shape since it is the driving force of the particle growth and coarsening.

**Figure 1:**
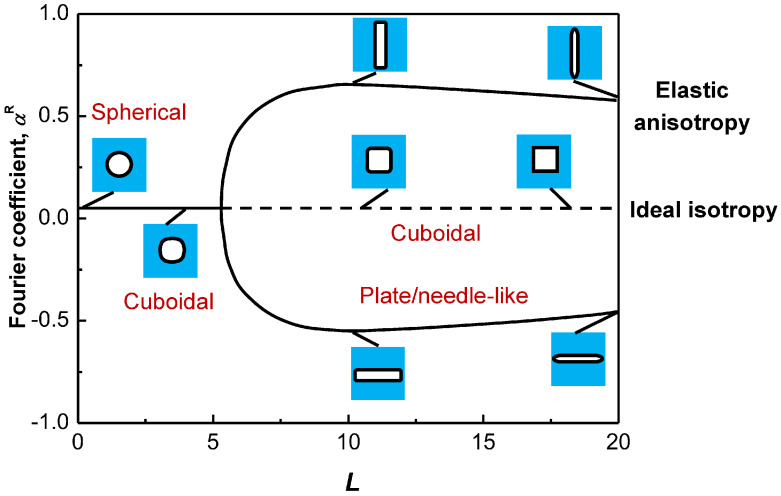
Particle morphology evolution with the characteristic parameter *L*, in which the vertical axis is the Fourier coefficient *a^R^* to represent the energy of different particle shapes. It shows that the bifurcation from the four-fold symmetric cuboid to the two-fold symmetric shapes (plate or needle) occurs at a critical value (*L* = 5.6) [33].

The relative importance of the elastic energy and the interfacial energy can be evaluated through the characteristic parameter *L* [32,33], i.e., *L* = *ε*_2_*C*_44_r/s, where *ε* is the lattice misfit between the particle and matrix phases, *C*_44_ is the elastic constant of the matrix, *r* is the average particle size, and *s* is the average specific interfacial energy. Figure 1 gives the morphology evolution of the particle with the parameter *L* [33], from which it is found that a small *L* usually corresponds to a spherical particle, which can transform to ellipsoidal or cuboidal shape when *L* increases. At a much larger *L*, the fourfold symmetry of cuboidal particles will be broken and some low symmetric shapes, like plates or needles, will begin to appear due to the elastic anisotropy. In addition, when the particle sizes are comparable, the lattice misfit *ε* will play the key role in determining the particle shape, since the parameter *L* is proportional to both the lattice misfit *ε* and the particle size *r*. Apparently, the particle morphology has a profound effect on the mechanical properties of alloys, which will be discussed in the following diverse alloy systems, respectively.

## 3. Precipitation Strengthening Mechanisms

The precipitation strengthening mechanisms can be divided into two categories [1,2,3], the dislocation shearing mechanism and the Orowan dislocation bypassing mechanism, depending on the interaction between moving dislocations and precipitates. The one leading to a smaller strength increment is the operative mechanism. The dislocation-shearing mechanism is generally active when the precipitates are coherent with the matrix, and the particle size is small, while the Orowan bypassing mechanism dominates when the coherent particle size exceeds a critical value or when the particles are incoherent with the matrix. For the shearing mechanism, three factors contribute to the increase in yield strength, coherency strengthening (Δ*σ_CS_*), modulus mismatch strengthening (Δ*σ_MS_*), and order strengthening (Δ*σ_OS_*). The former two (Δ*σ_CS_* and Δ*σ_MS_*) occur before the dislocation shears the particle and the latter (Δ*σ_OS_*) during shearing. Thereof, the larger value of (Δ*σ_CS_* + Δ*σ_MS_*) or Δ*σ_OS_* is expected to be the total strength increment from the shearing mechanism. The equations available to calculate these strength increments caused by both dislocation shearing and bypassing are as follows [34,35,36,37,38,39]:

(1) $$\Delta \sigma_{CS}=M\times \alpha_{\varepsilon }\times {\left(G\varepsilon_{c}\right)}^{\frac{3}{2}}\times {\left(\frac{rf}{0.5Gb}\right)}^{\frac{1}{2}}\;$$

(2) $$\Delta \sigma_{MS}=M\times 0.0055{\left(\Delta G\right)}^{\frac{3}{2}}\times {\left(\frac{2f}{G}\right)}^{\frac{1}{2}}\times {\left(\frac{r}{b}\right)}^{\frac{3m}{2}-1}\;$$

(3) $$\Delta \sigma_{OS}=M\times 0.81\times \frac{\gamma_{apb}}{2b}\times {\left(\frac{3\pi f}{8}\right)}^{\frac{1}{2}}\;$$

(4) $$\Delta \sigma_{orowan}=M\times \frac{0.4Gb}{\pi \sqrt{1-v}}\times \frac{\ln(2\sqrt{\frac{2}{3}}r/b)}{\lambda_{p}},\;\lambda_{p}=2\sqrt{\frac{2}{3}}r(\sqrt{\frac{\pi }{4f}}-1)\;$$

where *M* = 2.73 for BCC structure and *M* = 3.06 for FCC structure (Taylor Factor) [1], *α_ε_* = 2.6 (a constant) [35,36], *m* = 0.85 (a constant) [37,38], *ε*_c_ = 2*ε*/3 [2,35,36], the constrained lattice misfit. *G* and Δ*G* are the shear-modulus of the matrix and the shear modulus mismatch between precipitates and matrix, respectively; *b* is the Burgers vector; *r* and *f* are the average size and the volume fraction of precipitates, respectively; γ*_apb_* is the anti-phase boundary energy of precipitates; *v* is the Poisson ratio; and *λ_p_* is the inter-precipitate spacing.

**Figure 2:**
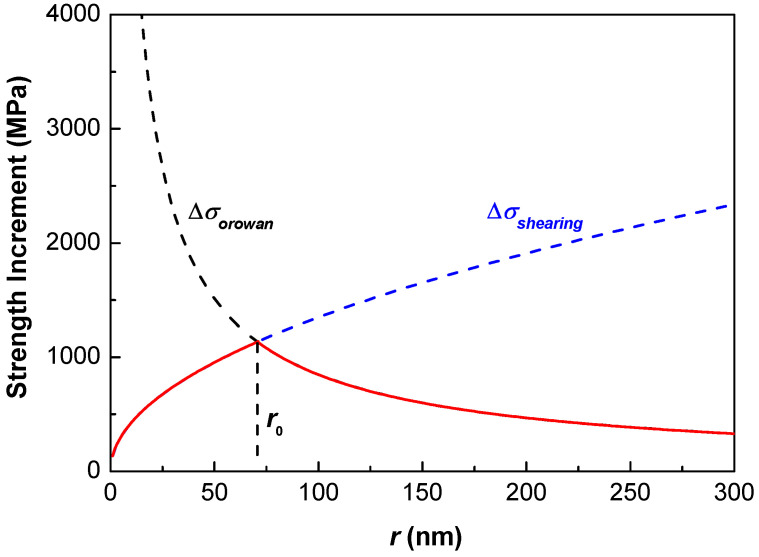
The variation tendency of yield strength increment with the particle size [1,2,3], in which the strength increments caused by dislocation shearing mechanism (Δ*σ_shearing_*) and bypassing mechanism (Δ*σ_orowan_*) are shown, and the maximum increment reaches at a critical particle size *r*_0_.

Since the shearing and bypassing mechanisms occur concurrently and are independent to each other, the strengthening is determined by the smaller of Δ*σ_shearing_* or Δ*σ_orowan_*. In other words, the softer mechanism initiates the plastic deformation. Ideally, the largest yield strength increment could be reached when Δ*σ*_shearing_ = Δ*σ_orowan_* at a critical particle size *r*_0_ with a fixed *f* [1]. Figure 2 shows the variation tendency of the yield strength increment with the particle size by competing the dislocation shearing and bypassing mechanisms, in which the maximum strength increment reaches at the critical *r*_0_ when the volume fraction *f* is fixed.

## 4. Precipitate Morphology and Precipitation Strengthening in CCAs

In this section, the precipitation behavior and precipitation strengthening effects in each CCA system are generalized in details. Typical alloy systems with precipitation strengthening include Ni-based superalloys, Al alloys, Mg alloys, Cu alloys, stainless steels, and high-entropy alloys. The overviews are elaborated as follows.

### 4.1. Ni-Based Superalloys

Ni-based superalloys exhibit the most outstanding mechanical properties (especially the creep-resistance), corrosion- and oxidation-resistant properties at elevated temperatures among all the conventional structural materials. Their excellent properties are benefited from their specially coherent microstructures of spherical or cuboidal L1_2_-γ′ nanoprecipitates into FCC-γ solid solution [4,5,6]. Especially the coherent precipitation of cuboidal L1_2_-γ′ particles in single-crystal superalloys is responsible for the necessary strength at much higher temperatures near to the melting point [40,41]. However, the single-crystal superalloys with similar compositions often possess different creep-resistant properties, even containing cuboidal γ′ precipitates with a comparable particle size. 

**Figure 3:**
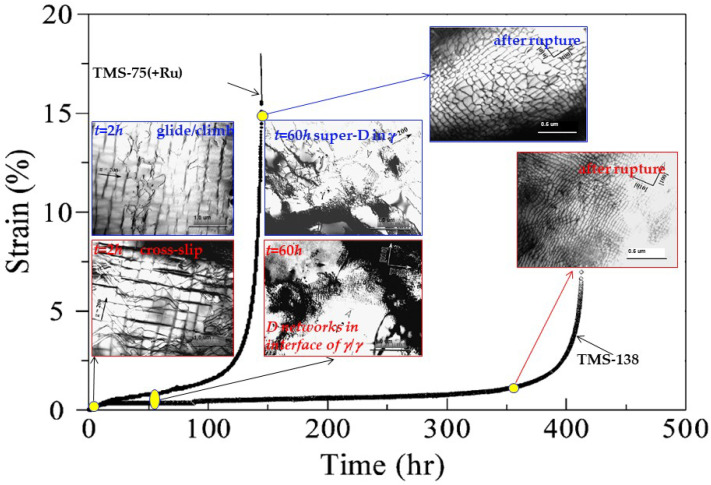
Creep curves at 1100 °C/ 137 MPa of TMS-75(+Ru) and TMS-138 superalloys, in which the microstructures at different creep stages (primary stage, steady state and after rupture) are also presented [40].

Figure 3 exhibits the creep curves at 1100 °C/137 MPa of TMS-138 (Ni-6Co-3Cr-3Mo-6W-6Al- 6Ta-0.1Hf-5Re-2Ru, wt.%) and TMS-75(+Ru) (Ni-12Co-3Cr-2Mo-6W-6Al-6Ta-0.1Hf-5Re-1.5Ru, wt.%) alloys, in which the microstructural evolutions during the creep process are also shown [40]. Both superalloys have similar compositions with a minor difference in the amounts of Co, Mo, and Ru. The particle sizes of cuboidal γ′ nanoprecipitates in these two alloys are comparable, being *r* = 230 ± 30 nm (TMS-138) and *r* = 245 ± 25 nm (TMS-75(+Ru)), respectively, with a volume fraction of about *f* = 65% in experiments. But the lattice misfit *ε* between γ and γ′ phases are different in both alloys, being *ε* = −0.33% in TMS-138 and *ε* = −0.16% in TMS-75(+Ru) at 1100 °C, respectively, in which the lattice misfit is calculated with the equation of *ε* = 2(*a*_γ__′_ − *a*_γ_)/(*a*_γ__′_ + *a*_γ_) (*a*_γ__′_ and *a*_γ_: the lattice constants of γ′ and γ phases, respectively). Remarkably, TMS-138 possesses a longer creep life and a lower minimum creep rate, which is attributed to its larger γ/γ′ lattice misfit *ε*. Specifically, in the primary creep stage, such as the time *t* = 2 h, the larger misfit stress caused by the larger *ε* in TMS-138 drives the loops of matrix dislocations to move by cross-slip through the matrix channels, while in TMS-75(+Ru), the dislocations move by climbing around the γ′ cuboids due to the insufficient driving force by the relatively-smaller *ε* (as seen in the microstructures in Figure 3). With increasing creep time to *t* = 60 h, there are many superdislocations in γ′ cuboids in TMS-75(+Ru), while many perfect dislocation networks on the γ/γ′ interface are formed in TMS-138, which can effectively prevent the gliding dislocations in the γ channels from cutting the rafted γ/γ′ structure. More importantly, the larger lattice misfit can result in the denser γ/γ′ interfacial dislocation networks. Clearly, the dislocation networks are much denser in TMS-138 after rupture, which is the key for the small minimum creep rate.

**Figure 4:**
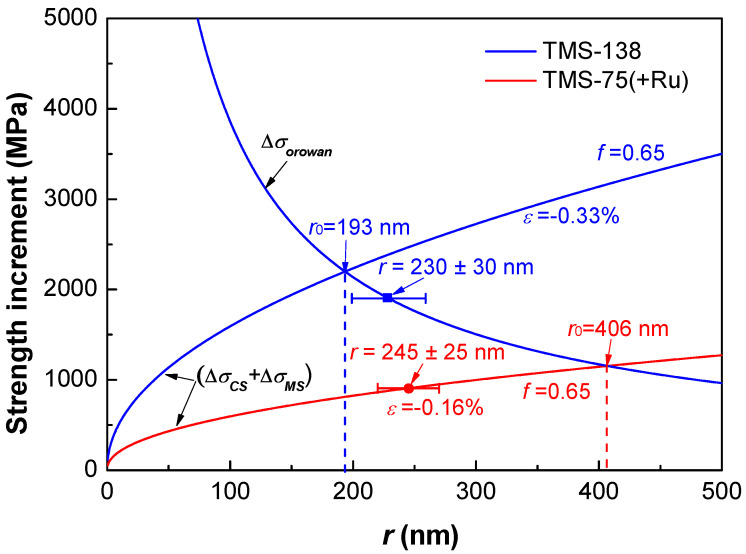
The variation tendency of (Δ*σ_CS_* + Δ*σ_MS_*) and Δ*σ_orowan_* with the particle size *r* of TMS-138 and TMS-75(+Ru) superalloys, in which the optimal particle size *r*_0_ from the calculation and the experimentally-measured *r* are also marked for each alloy.

Not only the precipitate morphology, but also the strengthening effect of coherent precipitation are closely related to the lattice misfit between the ordered phase and solid-solution phase. We calculated the yield strength increments given by shearing and bypassing mechanisms according to the Equations (1)–(4) with the particle size *r*, in which the dominant (Δ*σ_CS_* + Δ*σ_MS_*) presents the strength increment caused by the shearing mechanism. The used parameters for strength increment calculations are *M* = 3.06, *α_ε_* = 2.6, *G* = 81 GPa, Δ*G* = 4 GPa, *m* = 0.85, *b* = 0.254 nm, *γ_apb_* = 0.12 J/m^2^, and *v* = 0.35, respectively. Figure 4 gives the variation tendencies of (Δ*σ_CS_* + Δ*σ_MS_*) and Δ*σ_orowan_* as a function of the particle size *r* for TMS-138 and TMS-75(+Ru) superalloys. It was found that the optimal particle size *r*_0_ corresponding to the maximum strength increment given by the theoretical calculation in TMS138 is *r*_0_ = 193 nm, which is consistent with the experimental size of *r* = 230 ± 30 nm. While the experimental particle size (*r* = 245 ± 25 nm) is far away from its optimal *r*_0_ = 406 nm in TMS-75(+Ru), indicating that the strengthening effect does not reach the maximum. Hence, the TMS-138 superalloy exhibits a much higher strength and a better creep resistance due to a larger lattice misfit. Therefore, in the case of coherent precipitation, the control of the lattice misfit between the ordered phase and its parent solid solution is significant to develop high-performance CCAs.

### 4.2. Al-Based Alloys

**Figure 5:**
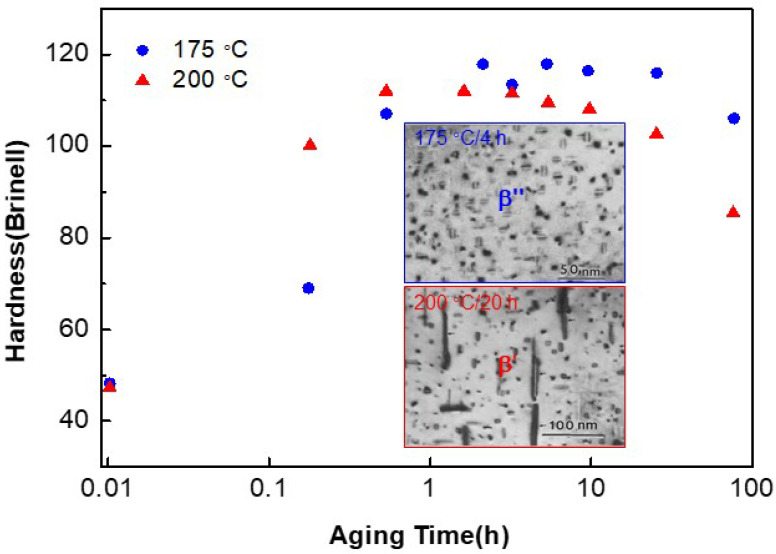
Variation of hardness of the 6061 Al alloy with the aging time at both 175 °C and 200 °C, in which the microstructures at peak aging and over aging are also shown [49].

Al alloys have been used widely as engineering structural materials due to their high specific strength, among which the high-strength Al-Zn-Mg-Cu series of alloys (7000 series) are extensively applied into aeronautical fields [42,43,44,45,46]. The Al-Cu binary system is a well-studied precipitation-strengthening system, since it forms the basis for many types of age-hardening alloys with technological importance [47]. The precipitation sequence during the aging process, Al SS → G.P. zone → θ″-Al_3_Cu → θ′-Al_2_Cu → θ-Al_2_Cu, was often taken as a model for describing the fundamentals of precipitation strengthening. The coherent Guinier-Preston (G.P.) zone consisting of a single layer of pure Cu atoms was firstly precipitated from the FCC-Al solid solution (SS) matrix. Then, Al-Cu clusters with a stoichiometrical Al_3_Cu (θ″) were formed, which is also coherent with the FCC matrix. It could transform into the metastable θ′-Al_2_Cu phase with a body-centered-tetragonal structure, which is the main strengthening phase, but semi-coherent with the matrix. Finally, the metastable θ′-Al_2_Cu would transform into the equilibrium tetragonal θ′-Al_2_Cu phase, which is incoherent with the matrix [48]. In this case, the coherent relationship between the precipitated phase and the matrix could be destroyed with prolonging the aging time, which eventually leads to the formation of coarse precipitates, as a final result of weakening the strengthening effect, compared with the coherent fine precipitates. Figure 5 shows the effect of the particle morphology on the hardness variation with the aging time of Al-0.8Mg-0.79Si (wt.%, 6061) alloy [49]. The peak hardness reaches at 175 °C aging for 4 h, corresponding to the semi-coherent precipitation of the needle β″ (a monoclinic structure with a stoichiometrically MgSi) nanoparticles with a size of about 10~15 nm. Once the needle β″ nanoparticles are transformed to relatively-coarse rod β′ particles (a hexagonal structure with a stoichiometrically Mg_1.7_Si) at 200 °C aging for 20 h, the hardness will decrease due to the incoherency of β′ and the matrix [49].

**Figure 6:**
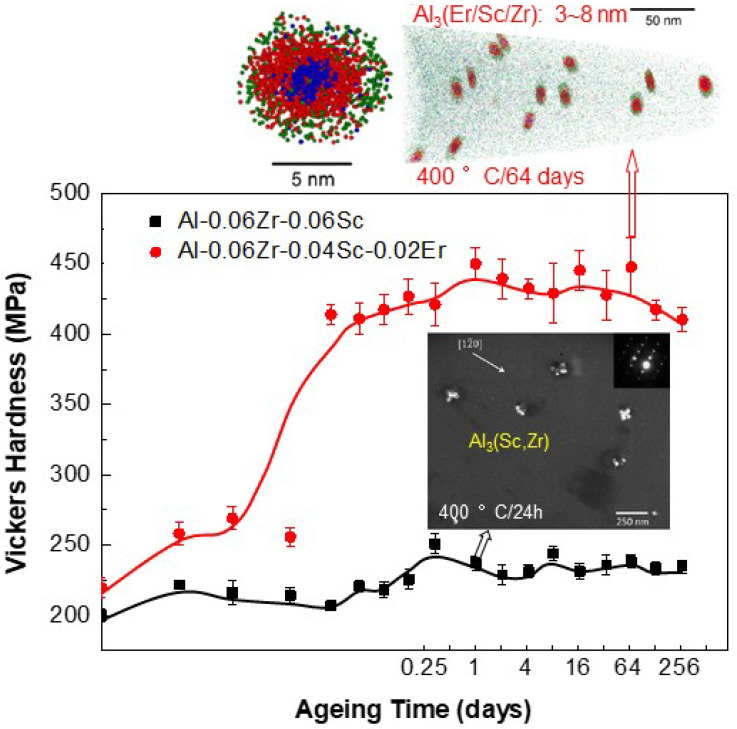
Variation of Vickers hardness of Al-Zr-Sc-Er alloys with the aging time at 400 °C, in which the precipitate morphologies of coherent Al_3_(Sc,Zr) after aging 24 h and Al_3_(Sc,Zr,Er) after aging 64 days are also presented [50].

Recently, the coherent precipitation of ordered L1_2_-Ni_3_M (M = Sc, Er, Zr, etc.) in the disordered FCC matrix of Al-Zr-Sc-Er alloy systems has attracted more attention since it can provide significant strengthening to a temperature of about 300 °C. Such Al alloys are excellent candidates for some high-temperature automotive and aerospace applications [50,51,52]. Supersaturated Al-Sc binary alloys generally possess high strength due to the coherent precipitation of L1_2_-Al_3_Sc nanoparticles [53,54]. Based on it, the addition of Zr can form coarsening-resistant L1_2_-Al_3_(Sc,Zr) lobed-cuboids consisting of a Sc-enriched core surrounded by a Zr-enriched shell in Al-0.06Sc-0.06Zr (wt.%) alloy [45,55] (as seen in Figure 6). More interestingly, the Er further substitution for Zr can form spheroidal Al_3_(Sc,Zr,Er) nanoprecipitates with a core/double-shell structure consisting of an Er-enriched core surrounded by a Sc-enriched inner shell and a Zr-enriched outer shell in the Al-0.06Sc-0.04Zr-0.02Er (wt.%) alloy (Figure 6), which are stable and difficult to be coarsened even at a higher temperature of 400 °C for 64 days [42]. Resultantly, it is due to the particle size that renders the two alloys with a remarkable difference in microhardness, as shown in Figure 6. The particle size of lobed-cuboidal Al_3_(Sc,Zr) precipitates is about 25 nm in the former alloy, while the spheroidal Al_3_(Sc,Zr,Er) nanoprecipitates with a particle size of 3~8 nm result in a drastic improvement of the alloy strength, from a peak harness of 243 MPa in the former alloy to 451 MPa in the later one at 400 °C aging. Therefore, more and more interests have been focused on the coherent precipitation in Al alloys to develop new light-weight materials that can be applied in high-temperature environments (>300 °C).

### 4.3. Mg-Based Alloys

**Figure 7:**
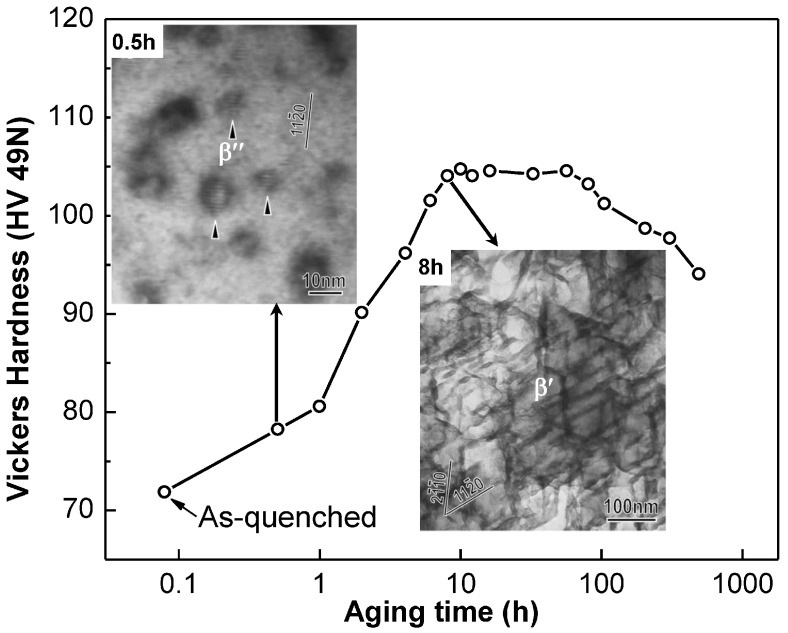
Variation of Vickers hardness of the Mg-15Gd-0.5Zr alloy with the aging time at 250 °C, in which the morphologies of coherent β″ and β′ precipitates are also shown [72,73].

Mg alloys with a close-packed-hexagonal (HCP) matrix are the lightest among all the commonly-used structural materials and have great potentials for application in the automotive, aircraft, aerospace, and electronic industries [56]. Their useful mechanical properties were generally achieved via age-hardening process to form high-strength precipitates, which is similar to the precipitation in Al alloys. Actually, it is more difficult to keep the coherency between the precipitates and the matrix due to the HCP structure of the matrix. The structure, morphology, and orientation of precipitates, precipitation sequence, and hardening response in various Mg alloy systems have been generalized, in which the effects of precipitate shapes on strengthening and the rational design of microstructures for higher strengths were also emphasized [57,58]. For the most widely-used Mg-Al-based alloys, such as AZ91 (Mg-8.7Al-0.7Zn-0.1Mn, wt.%), the final stable incoherent β-Mg_17_Al_12_ phase with a BCC structure precipitates directly from the supersaturated HCP-Mg solid solution without any coherent G.P. zones, which could not result in an appreciable strengthening response due to the existence of relatively-coarse plate/lath-like β particles [59,60,61]. For most of the high-strength Mg alloy series, the precipitation usually follows the sequence of Mg SS → G.P. zone → coherent metastable phase → incoherent stable phase [62,63,64,65,66,67,68,69,70,71]. A typical Mg-Gd(-Y)-based Mg-15Gd-0.5Zr (wt.%) alloy was taken for an instance [72]. Firstly, the coherent G.P. zone precipitated from the HCP-Mg solid solution at the initial stage of aging at 250 °C. Then the metastable β″-Mg_3_Gd phase with an ordered DO_19_ structure of the HCP solid solution appeared after aging for 0.5 h, which keeps the perfect coherent orientation with the matrix and exhibits a hexagonal prism morphology. Another metastable orthorhombic β′-Mg_3_Gd phase with a lenticular particle morphology would substitute for the β″ when the aging time increased to 8 h, resulting in a peak strengthening. Figure 7 shows the microhardness variation with the aging time, from which an obvious improvement of microhardness is attributed to the precipitation of metastable β″ and β′ phases. Further prolonging the aging time, β′ will transform into a FCC β_1_-Mg_3_Gd with a DO_3_-L2_1_ structure, and then to the final stable β-Mg_5_Gd with a FCC structure, which can weaken the strengthening response due to the plate-like morphology of β_1_ and β particles [72,73].

**Figure 8:**
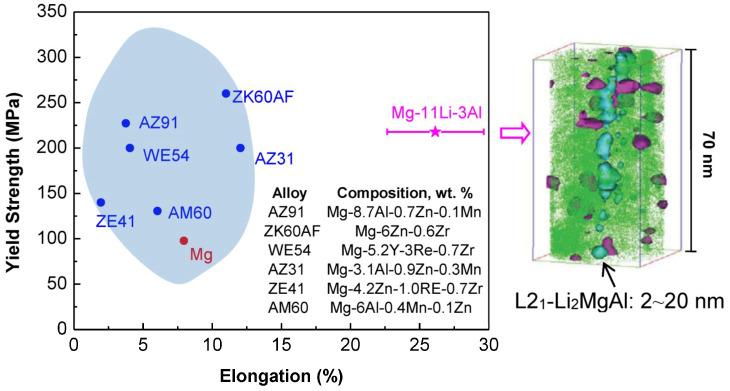
Comparison of the mechanical properties (yield strength and elongation) of the BCC-based Mg-11Li-3Al alloy in traditional HCP Mg alloys, in which the spherical L2_1_ nanoparticles of the former BCC Mg alloy are also presented [74].

It is emphasized that the DO_3_-L2_1_ β_1_ phase often exists in many high-strength Mg alloys, which is a highly-ordered superstructure of the BCC solid solution, consisting of eight BCC unit cells. If the HCP-Mg matrix is changed to a BCC structure, the coherency will be achieved between the L2_1_ phase and BCC-Mg solid solution. It is fascinating that the ordered L2_1_-Li_2_MgAl phase was coherently-precipitated into the BCC-Mg matrix in the recently-reported Mg-11Li-3Al (wt.%) alloy, which renders the alloy with high strength, good ductility, and excellent corrosion resistance [74]. Figure 8 compared the room-temperature mechanical tensile properties (yield strength and elongation to fracture) of Mg-11Li-3Al alloy with several traditional Mg alloys. It is found that this BCC-based alloy exhibits not only a higher strength, but also a much better ductility with an elongation to fracture of about 27%, which is attributed to the coherent precipitation of spherical L2_1_ nanoparticles with a particle size of 2~20 nm (as shown in Figure 8).

### 4.4. Cu-Based Alloys

**Figure 9:**
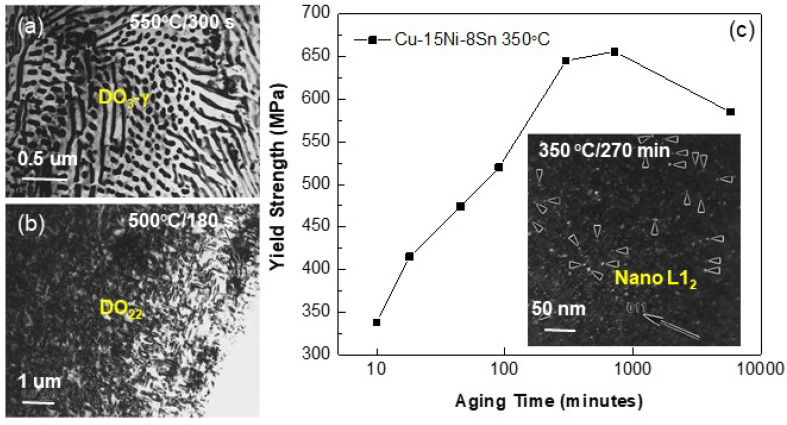
Morphologies of ordered DO_3_-(**a**), DO_22_-(**b**), and L1_2_-(Cu,Ni)_3_Sn precipitates (**c**), as well as the variation tendency of the tensile yield strength of Cu-15Ni-8Sn alloy with the aging time at 350 °C (**c**) [77,78].

The precipitates in various Cu alloys generally show different phase structures, in which the coherent precipitation of metastable ordered L1_2_ in the FCC-Cu solid solution matrix can appear in the commonly-used Cu-Ni-Sn alloy system [75,76,77]. The model alloy is the Cu-15Ni-8Sn (wt.%, C72900), in which the (Cu,Ni)_3_Sn precipitates have four crystal structures, FCC-DO_3_, tetragonal DO_22_, ordered L1_2_, and orthorhombic δ. The phase evolution sequence from high to low temperatures is similar to that in aged Al alloys for different times. When aging at above 550 °C, discontinuous and intragranular DO_3_-γ precipitates (plate-like shape) appeared in the FCC matrix, as seen in Figure 9a. Spinodal decomposition often occurred during the early stage of the decomposition below ~500 °C, followed by the DO_22_ ordering (as seen in Figure 9b) and then L1_2_ ordering (the inset of Figure 9c) [77]. With decreasing aging temperature, spherical L1_2_ nanoparticles could be coherently-precipitated into the FCC matrix, compared with the rod-like DO_22_ precipitates. The particle morphology can affect the mechanical property of this alloy, as seen in Figure 9c, being the variation tendency of the tensile yield strength with the aging time [77,78,79]. It was found that the coherent precipitation of spherical L1_2_ nanoprecipitates corresponds to the highest strength.

### 4.5. Fe-Based Stainless Steels

**Figure 10:**
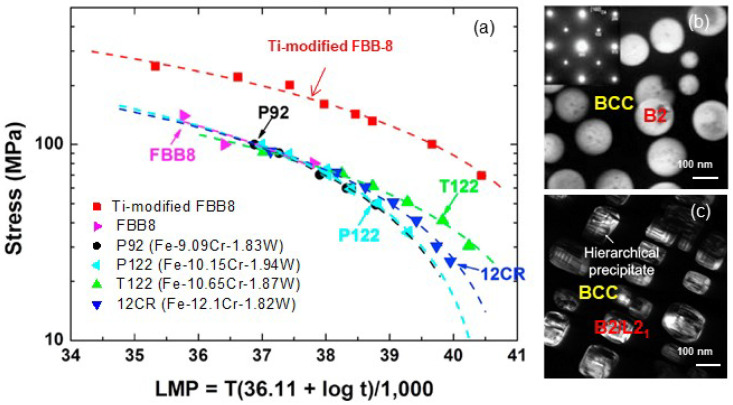
(**a**) Larson-Miller parameters (LMP) for FBB8, Ti-modified FBB8, and several conventional steels (P92, P122, T122, and 12CR) [89]; (**b**,**c**) morphologies of spherical B2 nanoprecipitates in FBB8 [12] and cuboidal L21 nanoprecipitates in Ti-modified FBB8 [89,90], respectively.

It is well known that the most common strengthening precipitates are carbides, being Cr_23_C_6_ and MC (M = Nb, Ti, V, etc.) [7,8]. For some special stainless steels (SSs), there also exist other kinds of precipitates, such as Laves phases (Fe_2_M), Ni_3_M, B2-NiAl, σ-FeCr, and Z-CrNbN, to strengthen the FCC or BCC matrix [7]. For instance, austenitic SSs for the use in high-temperature (600~800 °C) and oxidation environment are generally strengthened by MC, Cr_23_Cr_6_, Z, Fe_2_M, or B2-NiAl [80,81]. However, it is noted that these phases are not coherent with the FCC austenite matrix, which can lead to the coarsening of second phase precipitates, as a final result of softeness or embrittlement (the latter mainly caused by the σ phase). Very interestingly, another kind of high-temperature ferritic SSs with a coherent microstructure of ordered B2 phase precipitation into BCC matrix have been developed [12,13,82,83,84,85,86,87,88]. As shown in Figure 10a, the Fe-6.5Al-10Ni-10Cr-3.4Mo-0.25Zr-0.005B (wt.%, FBB8) alloy exhibits a prominent creep resistance at 700 °C, better than the conventional P92, P122, T122, and 12CR steels [12,84]. It is attributed to the coherent precipitation of spherical B2 nanoparticles into BCC ferritic matrix (Figure 10b), similar to that in FCC Ni-based superalloys [5]. The further addition of 2 wt.% Ti into FBB8 induces another ordered L2_1_-Ni_2_AlTi phase of the BCC solid solution, forming a coherent microstructure with cuboidal B2/L2_1_ hierarchical precipitates (Figure 10c) [89,90]. We calculated the lattice misfit between BCC and B2/L2_1_ phases with the formulas of *ε* = 2(*a*_B2_ − *a*_BCC_)/(*a*_B2_ + *a*_BCC_) and *ε* = 2(*a*_L21_ − 2*a*_BCC_)/(*a*_L21_ + 2*a*_BCC_), in which *a*_B2_, *a*_L21_, and *a*_BCC_ are the lattice constants of B2, L2_1_, and BCC solid solution phases, respectively. It was found that the cuboidal precipitation of B2/L2_1_ phases mainly resulted from the larger lattice misfit of *ε* = 0.7% between the BCC and L2_1_ phases in the Ti-modified FBB8, while the smaller value of *ε* = 0.06% promotes the formation of spherical B2 precipitates in FBB8. More importantly, it is due to the cuboidal precipitation that further improves the creep-resistant property of the Ti-modified FBB8 alloy at 700 °C (Figure 10a), possessing a better creep life than FBB8.

**Figure 11:**
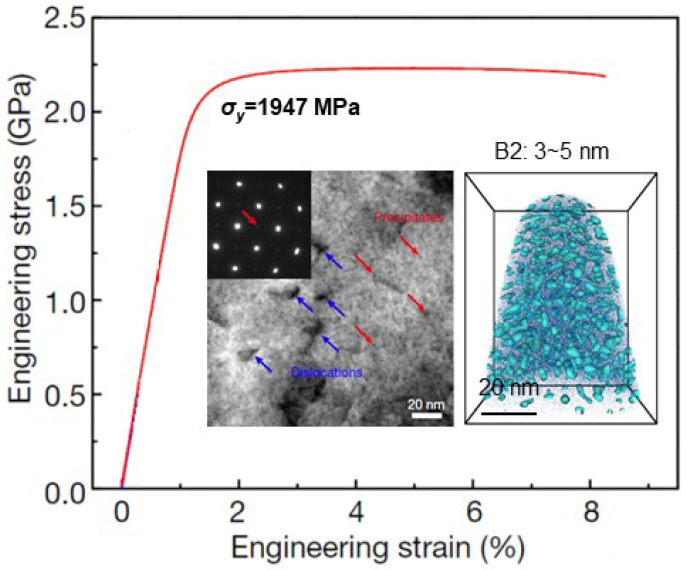
Tensile engineering stress-strain curve of the aged Fe-17Ni-6.2Al-2.3Mo-0.48Nb- 0.37C-0.05B steel, in which the microstructures of B2 nanoparticles with a size about 3~5 nm and dislocations are also shown [9].

Moreover, the precipitation of intermetallic compound Ni_3_M in the BCC martensite matrix can make maraging stainless steels with a much higher strength (tensile yield strength of σ_y_ = 1.2~1.5 GPa) [91,92,93,94]. Very recently, the coherent precipitation of spherical B2 nanoparticles with a particle size of 3~5 nm, rather than the Ni_3_M, in the BCC martensite rendered a Fe-17Ni-6.2Al-2.3Mo-0.48Nb-0.37C-0.05B (wt.%) steel with a superhigh strength (σ_y_ > 1.9 GPa) [9], as shown in Figure 11. It is attributed to the smaller lattice misfit (*ε* = 0.17%) that permits the dislocations cutting through the B2 nanoparticles, in which the high anti-phase boundary (APB) energy can promote the ordering strengthening by increasing the dislocation shear resistance in the particles.

### 4.6. High-Entropy Alloys

High-entropy alloys (HEAs) with equimolar or near-equimolar mixing of multiple elements have been found in diverse alloy systems and have attracted more attention due to their interesting properties and associated scientific understandings. Especially, CoCrFeNi-based HEAs [95,96,97,98,99,100,101,102,103,104], composed of late transition metals (LTMs), were widely investigated due to their exceptional mechanical properties and potential industrial applications. For instance, the single FCC CoCrFeNiMn HEA displays an excellent damage tolerance with higher tensile strength and remarkable fracture toughness than traditional engineering stainless steels at cryogenic temperatures down to −196 °C, which resulted from the microstructural diversity caused by the twinning-induced plasticity (TWIP) effect [98].

A further addition of Al or Ti into these LTMs-based HEAs can produce phase transformations [105,106,107,108,109,110,111,112,113,114,115,116,117,118,119,120,121,122,123,124,125,126,127,128,129,130,131], and then lead to microstructural diversities due to the strong interactions between Al/Ti and LTMs, resulting in an enhancement of strength. It is primarily attributed to the coherent precipitation of intermetallic phases, such as L1_2_-Ni_3_Al from the FCC matrix [125,126], and B2-NiAl [110,118,119,120,121,122,123,124] and L2_1_-Ni_2_AlTi [107,108,109] from the BCC matrix. As example, the (NiCoFeCr)_94_Ti_2_Al_4_ (at.%) HEA possesses a special coherent microstructure with fine spherical L1_2_-(Ni_3_(Al,Ti)) nanoprecipitates in the FCC matrix, resulting in a significant strength improvement with a yield strength over 1 GPa [125]. A newly-developed kind of high entropy Ni-based alloys, such as Ni_48.6_Al_10.3_Co_17_Cr_7.5_Fe_9.0_Ti_5.8_Ta_0.6_Mo_0.8_W_0.4_ (at.%), exhibit a higher high-temperature hardness resulted from the γ′-L1_2_ precipitation-strengthening of γ-FCC matrix, where the coherent γ/γ′ microstructure can be thermodynamically stable after aging from 700 °C to 1100 °C for at least 500 h [126]. It is noted that a small amount of Al addition in HEAs (e.g., Al_0.3_FeCoNiCr [115,116,117] and Al_8_Co_17_Cr_17_Cu_8_Fe_17_Ni_33_ [127]) generally renders spherical L1_2_-Ni_3_Al particles precipitated in the FCC matrix, resulting in high strength and good ductility, similar to that in Ni-based superalloys [5,132]. With increasing Al content, not only the FCC matrix of Al_x_FeCoNiCr series of HEAs transforms into the BCC phase, but also the precipitates change from L1_2_-Ni_3_Al to B2-NiAl, as a result of an enhancement of strength drastically [114,115,116,117,118]. In addition, fixing Al content, the mutation of transition metals can also change the phase transition from FCC to BCC, as evidenced by the two HEAs of the FCC-based Fe_36_Co_21_Cr_18_Ni_15_Al_10_ (at.%) and the BCC-based Fe_36_Mn_21_Cr_18_Ni_15_Al_10_ (at.%) HEA with cuboidal B2 nanoprecipitates [109,123]. Furthermore, a minor addition of Ti (4 at.%) into these two HEAs leads to the formation of L2_1_-Ni_2_AlTi phase, rather than the B2, but the L2_1_ precipitates exhibit different morphologies, being plate-like shape in the former alloy and cuboidal shape in the latter, respectively [109].

Experimentally, it is difficult to obtain cuboidal or spherical morphology of coherent B2 or L2_1_ precipitates in BCC-based HEAs, which is primarily attributed to a large lattice misfit between BCC and B2 phases caused by the large composition difference. Thus, a weave-like microstructure of BCC and B2/L2_1_ always occurred in these BCC-based HEAs, since it is sensitive to the Al/Ti content, such as the AlFeCoNiCr HEA, leading to a serious brittleness [114]. Massive efforts have been done to research for the cuboidal or spherical B2/L2_1_ precipitation in various systems through adjusting both Al and transition metals [107,108,109,118,119,120,121,122,123,124,128,129]. It is fascinating that spherical or cuboidal B2/L2_1_ nanoprecipitates are coherently-existed not only in Al/Ti-LTM HEAs (e.g., Fe_34_Cr_34_Ni_14_Al_14_Co_4_ at.% [121]), but also in refractory HEAs consisted of Al and early transition metals [111,128,129]. For instance, the coherent precipitation of spherical B2 nanoparticles in BCC matrix improves the room- temperature compressive ductility on a large extent of refractory Al_0.5_NbTa_0.8_Ti_1.5_V_0.2_Zr HEA, while maintains high yield strength at both room and elevated temperatures [129]. 

Table 1 lists the mechanical properties (yield strength *σ*_y_ and ductility *δ*) at room and elevated temperatures of some precipitation-strengthened HEAs, including L1_2_-strengthed FCC-based HEAs and B2/L2_1_-strenthened BCC-based HEAs. The mechanical properties of commercial boiler steel HR3C (Fe_54.73_Cr_24.01_Ni_20.6_C_0.05_Nb_0.37_N_0.24_, wt.%) [133,134], newly-developed Ti-modified FBB8 ferritic stainless steel [89,90], and commercial Ni-based polycrystalline superalloy Inconel 718 (Ni_53_Fe_18.5_Cr_19_Nb_5.1_Mo_3.0_Ti_0.9_Al_0.5_ wt.%) [135,136] were also listed in Table 1 for reference. Compared with the HR3C austenitic stainless steel strengthened by MC-type carbides and Z-NbCrN nanoparticles, the FCC-based HEAs containing coherent spherical L1_2_ nanoprecipitates generally exhibit higher yield strengths at both room and elevated temperatures, in which the particle size must exceed a certain value to ensure high strength according to the strengthening mechanism. In fact, the high-temperature strength has been improved in the newly-developed Ti-modified FBB8 ferritic stainless steel due to the coherent precipitation of cuboidal L2_1_ nanoparticles [89,90]. The higher room-temperature strengths of BCC-based HEAs with cuboidal B2/L2_1_ precipitation are comparable to that of Ni-based Inconel 718 superalloy. Especially for the Al-contained refractory HEAs [128,129], the yield strengths at both room and elevated temperatures (up to 1200 °C) are all much higher than that of Inconel 718, which will be softened at the temperature above 900 °C.

**Figure 12:**
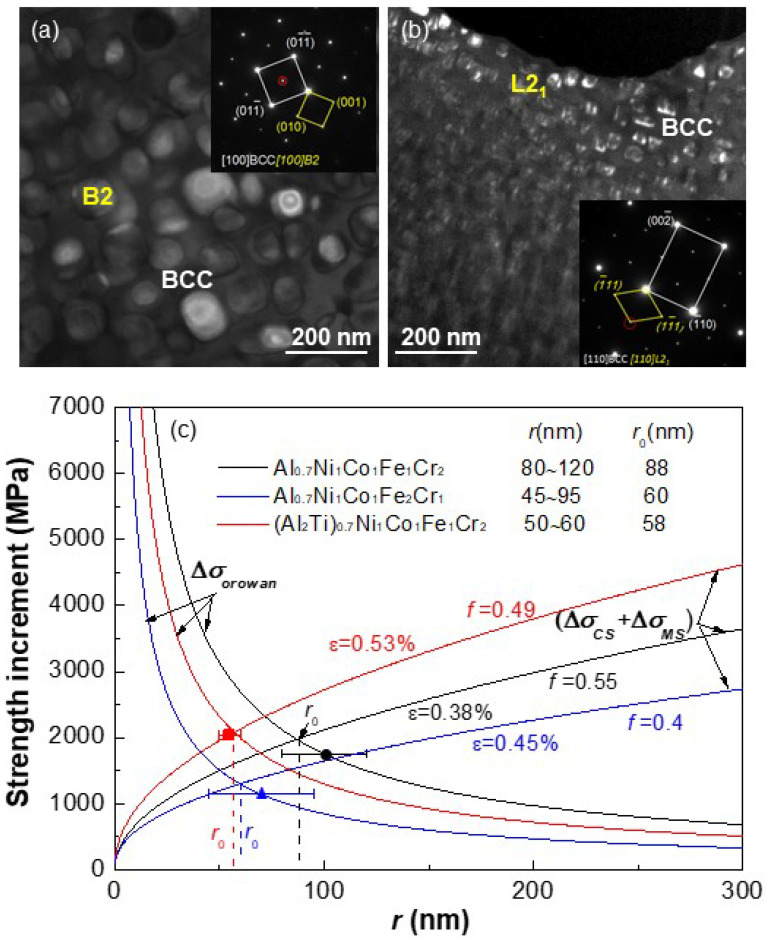
Computations of (Δ*σ_CS_* + Δ*σ_MS_*) and Δ*σ_orowan_* as a function of particle size *r* for the present Al_0.7_Ni_1_Co_1_Fe_1_Cr_2_, Al_0.7_Ni_1_Co_1_Fe_2_Cr_1_, and (Al_2_Ti)_0.7_Ni_1_Co_1_Fe_1_Cr_2_ HEAs (**c**), in which the morphologies of the coherent cuboidal B2 (**a**) and L2_1_ (**b**) precipitates are also presented [107,108,120]. The optimal particle size *r_0_* from the calculation and the experimentally-measured *r* are also marked for each alloy.

In our recent work, we obtained spherical or cuboidal B2 nanoprecipitates in the BCC matrix of Al-TM HEAs with the composition formula of Al_2_M_14_ (=Al_0.7_M_5_ in molar fraction), in which Al is fixed and M represents different mutations of Ni, Co, Fe, and Cr [119,120]. The Al_2_M_14_ was designed with the guide of a cluster formula approach through mutating the combinations of TMs, rather than the Al. It is noted that the addition of a much more amount of BCC stabilizers (Fe and Cr) can favor to the formation of the BCC/B2 structures without any FCC phase in alloys. More significantly, the cuboidal B2 nanoprecipitation in alloys with M = NiCoFe_2_Cr and M = NiCoFeCr_2_ is strongly attributed to a moderate lattice misfit (*ε* ~0.4%) between BCC and B2 phases, as seen in Figure 12a [120]. It is due to the cuboidal B2 nanoparticles in the BCC matrix that produces a prominent mechanical property with higher strength (*σ*_y_ = 1.1~1.7 GPa) and good ductility. Besides, these cuboidal B2 nanoprecipitates in these two alloys are very stable and could not be coarsened even after a long-time aging at 773 K for 1080 h [137]. In addition, the Ti substitution for Al in Al_2_M_14_ HEAs can change the phase structures of ordered precipitates, from B2-NiAl to L2_1_-Ni_2_AlTi. Furthermore, a minor amount of Ti substitution with a ratio of Al/Ti ≥ 2/1 can still keep the cuboidal morphology of L2_1_ nanoprecipitates for the achievement of a higher strength (*σ*_y_ = 1.8 GPa) due to a moderate lattice misfit (Figure 12b) [107,108]. Figure 12c shows the variation tendencies of (Δ*σ_CS_* + Δ*σ_MS_*) and Δ*σ_orowan_* with the particle size *r* in typical (Al,Ti)_2_M_14_ alloys at a fixed *f*. The used parameters for yield strength increment calculations are *M* = 2.73, *α_ε_* = 2.6, *G*_BCC_ = 83 GPa, *G*_B2_ = 80 GPa, *G*_L21_ = 73.6 GPa, *m* = 0.85, *b* = 0.254 nm, (*γ_apb_*)_B2_ = 0.25 J/m^2^, (*γ_apb_*)_L21_ = 0.04 J/m^2^, and *v* =0.3, respectively. The optimal particle size *r_0_*, corresponding to the largest strength increment, is calculated by the equation of (Δ*σ_CS_* + Δ*σ_MS_*) = Δ*σ_orowan_* in light of the precipitation strengthening mechanism. It is noted that when the particle size of B2 or L2_1_ nanoprecipitates exceeds a certain value (about 40 nm), the (Δ*σ_CS_* + Δ*σ_MS_*), rather than the Δ*σ_OR_*, will dominates the dislocation-shearing mechanism, compared with the phenomenon in the above-mentioned superhigh strength Fe-17Ni-6.2Al-2.3Mo-0.48Nb-0.37C-0.05B (wt.%) alloy [9]. More importantly, it can be demonstrated that the higher strength of (Al,Ti)_2_M_14_ HEAs is primarily attributed to the fact that the experimental particle size (50~120 nm) of B2 or L2_1_ cuboids is close to the optimal size *r*_0_ for the maximum strength increment from theoretical calculations. 

## 5. Thoughts on the Coherent Precipitation Strengthening

The coherent precipitation of ordered L1_2_-γ’ nanoprecipitates into the FCC-γ matrix renders the Ni-based superalloys with prominent mechanical properties at elevated temperatures close to melting points, which is hard to realize in other conventional alloy systems. The main reason is that in most conventional alloy systems, such as Al alloys, Mg alloys, and Cu alloys, the finally-stable precipitated phases are not the ordered superstructures of their parent solid solutions. In fact, the precipitation sequence of G.P. zones → metastable coherent ordered phases → stable non-coherent intermetallic phases often appears during the aging process, in which only the coherent precipitation corresponds to the peak strength. Therefore, many researchers have been exploring how to maintain the long-term stability of coherent precipitates through adjusting the amount of alloying elements or changing element species. Take the Mg alloys for instance. Based on the Mg-Al binary alloys without any ordered phases, the addition of a superlarge-size element Ca can form an ordered G.P. zone on the basal plane of the HCP matrix, i.e., clusters induced by Ca atoms, which can improve the creep resistance, but cannot enhance the strength [65]. So, in order to further improve the alloy strength, Mg-Gd-, Mg-Y-, and Mg-Nd-based alloys have been developed, in which several typical alloys, such as Mg-18.2Gd-1.9Ag-0.3Zr, Mg-6Y-4.9Zn, and Mg-10Gd-5.7Y-1.6Zn-0.7Zr (wt.%), possess a much higher ultimate tensile strength of above 400 MPa [57]. An obvious feature in these alloy systems is that the interplanar distance of G.P. zones (*d* = 0.37 nm) is getting closer to the lattice constant (*a*~0.32 nm) of the basal plane of HCP-Mg matrix, compared with that (*d* = 0.556 nm) in Mg-Al-Ca alloys. Hence, the much better coherency between the G.P. zones and the Mg matrix promotes the formation of ordered superstructure (DO_19_-Mg_3_(Gd,Nd)) of the HCP solid solution, resulting in a higher strength. From the viewpoint of alloying element species, it can be found that the key factor for the better coherency is that the super large-size elements (Gd, Y, etc.) and small-size elements (Zn, Ag, etc.) must be added simultaneously to balance the interplanar distance of G.P. zones close to the lattice constant of Mg matrix. Till recently, the HCP-Mg matrix was changed into the BCC-Mg matrix in Mg-Li-Al system, in which the coherent precipitation of ordered superstructure of L2_1_-Li_2_MgAl improves the tensile ductility, besides the higher strength of alloys [74]. It demonstrates sufficiently the important role of coherent precipitation in the development of high-strength structural materials.

In addition, in the case of coherent precipitation, the morphology of the coherent precipitates is also important to the mechanical properties of alloys, which has been identified by the presence of cuboidal L1_2_-γ′ nanoprecipitates in Ni-based single-crystal superalloys [5]. It is known that the precipitate morphology (shape and size) is primarily controlled by the lattice misfit between the ordered phase and its parent solid solution. A moderate lattice misfit for cuboidal nanoprecipitates should be achieved by mutating the lattice constants of these two phases simultaneously. For the γ/γ′ coherent microstructure, it is relatively easy to adjust the lattice misfit because there exists a relatively-small composition difference between γ and γ′ phases. In contrast, it is difficult to adjust the lattice misfit between the B2 or L2_1_ phase and the BCC matrix rationally in conventional alloy systems due to the relatively-larger composition difference between these two phases. In most cases, there often exhibits a weave-like microstructure induced by spinodal decomposition. The recent development of high-entropy alloys shows a bright insight to achieve the ideal (cuboidal) coherent microstructure since the phase compositions can be adjusted within a wide range in multi-principal alloy systems. Actually, all of the Ni-based single-crystal superalloys are concentratedly-complex alloys, generally containing more than ten elements, which favors the mutation of lattice misfit. So, some new BCC-based superalloys with superhigh strength and/or better creep resistance have been developed recently, which is derived from the coherent precipitation of spherical/cuboidal B2 or L2_1_ nanoprecipitates in the BCC matrix [9,89,90,108,120].

It is emphasized that whether the formation of coherent phases or the morphology of coherent precipitates is considered, both are closely related to the chemical composition. However, the rational matching of solute elements in the ordered phase and the solid solution was seldom considered when designing alloy compositions, since the solute distribution in the parent solid solution has not been clear until now. Actually, the local chemical structural units (chemical short range orders, CSROs) induced by solute elements are crucial to the stability of parent solid solution at high temperatures [138,139], which decides the precipitation of ordered phase during the aging or as-cast state. The recently-proposed cluster-plus-glue-atom model has defined such local chemical structure units in solid solutions [120,140,141,142], in which the cluster is the nearest-neighbor polyhedron centered by a solute atom having the strong interaction with the base solvent atoms to represent the strongest CSRO, and some other solute atoms (i.e., glue atoms) with weak interactions are certainly required to fill the space between the clusters to balance the atomic-packing density. Thus, a composition formula of [cluster] (glue atom) *x* (*x* being the glue-atom number) can be obtained from the cluster model. So, the phase compositions of ordered phase and their parent solid solution could be considered in light of the chemical structural units, respectively. Combined with the volume fraction of coherent precipitates, it would provide a new approach of composition design to develop high-performance compositionally-complex alloys with coherent precipitation.

## 6. Conclusions

In the present work, the precipitation behavior and precipitation strengthening in compositionally-complex alloys were generalized comprehensively, including high-performance conventional engineering materials (Ni superalloys, Al alloys, Mg alloys, Cu alloys, and stainless steels), and newly-developed high entropy alloys. The morphology evolution of second-phase particles and precipitation strengthening mechanism were introduced firstly. Then, the precipitation behaviors in diverse compositionally-complex alloy systems are illustrated, respectively. After discussing the relationship between the particle morphology and strengthening effectiveness, alloys with the coherent microstructure of the ordered phase precipitated in the disordered solid solution matrix were specially emphasized, since they exhibit prominent mechanical properties (superhigh strength/toughness and excellent high-temperature creep resistance). The universal feature existed in all compositionally-complex alloys is the coherent precipitation, which will be the most effective approach for the enhancement of alloy strength.

## Figures and Tables

**Table 1:** Data summary for some typical precipitation-strengthened HEAs, including alloy composition, matrix phase, particle size and morphology of precipitated phase at room temperature (RT), and mechanical properties (yield strength *σ*_y_ and ductility *δ*) at both room and elevated temperatures. The mechanical properties of commercial HR3C steel and Inconel 718 superalloy, as well as Ti-modified FBB8, are also listed for reference.

Alloys	Matrix Phase	Particle Size (nm) and Morphology of Precipitated Phase at RT	Mechanical Properties		
			Temp. (°C)	*σ*_y_ (MPa)	*δ* (%)
(NiCoFeCr)_94_Ti_2_Al_4_ (at.%) [125] (tensile test)	FCC	25 Spherical L1_2_	RT	1005	17
Ni_48.6_Al_10.3_Co_17_Cr_7.5_Fe_9.0_Ti_5.8_Ta_0.6_Mo_0.8_W_0.4_ (at.%) [126] (microhardness test)	FCC	287 Spherical L1_2_	RT	1230	-
			500	1128	-
			700	1056	-
			900	918	-
Fe_36_Co_21_Cr_18_Ni_15_Al_10_ (at.%) [123] (compressive test)	FCC	90 plate-like B2/BCC	RT	250	>50
			400	155	>50
			600	150	>50
Al_0.3_FeCoNiCr [117] (tensile test)	FCC	35 Spherical L1_2_	RT	160	60.8
Al_0.5_CoCuCrFeNi [113,131] (tensile test)	FCC	200~400 Spherical L1_2_	RT	1290	5.7
			600	425	2.8
			700	186	5.4
Al_8_Co_17_Cr_17_Cu_8_Fe_17_Ni_33_ (at.%) [127] (tensile test)	FCC	<20 Spherical L1_2_	RT	357	9
			500	315	0.7
Fe_36_Mn_21_Cr_18_Ni_15_Al_10_ (at.%) [123] (tensile test)	BCC	160 Cuboidal B2	RT	750	2.5
			400	640	20
			500	515	42
			600	310	55
Fe_35_Mn_20_Cr_17_Ni_12_Al_12_Ti_4_ (at.%) [109] (compressive test)	BCC	190 Cuboidal L2_1_	RT	1280	31
			400	1100	>50
			600	355	>50
Fe_35_Co_20_Cr_17_Ni_12_Al_12_Ti_4_ (at.%) [109] (compressive test)	BCC	65 Plate-like L2_1_	RT	1420	18
			400	1285	24
			600	795	>50
			800	285	>50
Al_0.7_NiCoFe_2_Cr [119] (tensile test)	BCC	50~90 Cuboidal B2	RT	1085	8.2
			650	454	6.4
			700	108	5.5
Al_0.7_NiCoFeCr_2_ [120] (compressive test)	BCC	80~120 Cuboidal B2	RT	1718	26.5
(Al_2_Ti)_0.7_NiCoFeCr_2_ [107] (compressive test)	BCC	50~60 Cuboidal L2_1_	RT	1808	35
AlMo_0.5_NbTa_0.5_TiZr [128] (compressive test)	B2	10~55 Cuboidal & plate-like BCC	RT	2000	10
			600	1870	10
			800	1597	11
			1000	745	>50
			1200	250	>50
Al_0.5_NbTa_0.8_Ti_1.5_V_0.2_Zr [129] (compressive test)	BCC	50 Spherical B2	RT	1345	38
			600	1423	16.2
HR3C Fe_54.73_Cr_24.01_Ni_20.6_C_0.05_Nb_0.37_N_0.24_ (wt.%) [132,133] (tensile test)	FCC	25~30 Spherical Z-NbCrN phase	RT	368	48
			650	180	46
Ti-modified FBB8 Fe_67.85_Al_6.5_Ni_10_Cr_10_Mo_3.4_Ti_2_Zr_0.25_B_0.005_ (wt.%) [89,90] (In-situ tensile test)	BCC	84~138 Cuboidal L2_1_	RT	1138 MPa, converted from *HV* value	
			700	230	7.5
Inonel 718 Ni_53_Fe_18.5_Cr_19_Nb_5.1_Mo_3.0_Ti_0.9_Al_0.5_ (wt.%) [134,135] (tensile test)	FCC	15~25 Spherical γ′-L1_2_ and γ′′-Ni_3_Nb	RT	1206	17.8
			600	1048	17.1
			700	979	20.5
			800	629	28.0
			900	262	83.0
